# Adjuvant Treatment in Pancreatic Cancer: Shaping the Future of the Curative Setting

**DOI:** 10.3389/fonc.2021.695627

**Published:** 2021-08-16

**Authors:** Annalisa Pappalardo, Emilio Francesco Giunta, Giuseppe Tirino, Luca Pompella, Piera Federico, Bruno Daniele, Ferdinando De Vita, Angelica Petrillo

**Affiliations:** ^1^Medical Oncology Unit, Ospedale del Mare, Naples, Italy; ^2^Division of Medical Oncology, Department of Precision Medicine, School of Medicine, University of study of Campania “L. Vanvitelli”, Naples, Italy

**Keywords:** PDAC - pancreatic ductal adenocarcinoma, biomarkers, ctDNA = circulating tumor DNA, gemcitabine, predictive factors, PARPi, target therapy, neoadjuvant chemotherapy

## Abstract

Pancreatic ductal adenocarcinoma (PDAC) is a lethal disease even in the early stages, despite progresses in surgical and pharmacological treatment in recent years. High potential for metastases is the main cause of therapeutic failure in localized disease, highlighting the current limited knowledge of underlying pathological processes. However, nowadays research is focusing on the search for personalized approaches also in the adjuvant setting for PDAC, by implementing the use of biomarkers and investigating new therapeutic targets. In this context, the aim of this narrative review is to summarize the current treatment scenario and new potential therapeutic approaches in early stage PDAC, from both a preclinical and clinical point of view. Additionally, the review examines the role of target therapies in localized PDAC and the influence of neoadjuvant treatments on survival outcomes.

## Introduction

Pancreatic ductal adenocarcinoma (PDAC) is a candidate for the second leading cause of cancer-related death in 2030, with a five-year survival rate of 5-7% (1). Surgical treatment with the goal of radical resection -tumor-free excision margins (so called “R0 resection”) is the only potentially curative approach for PDAC. However, only 15-20% of patients with PDAC have localized and potentially resectable disease at diagnosis (2).

In recent years, radiological criteria were developed in order to define tumor resectability and to improve the selection of patients able to receive a curative surgical approach. In detail, according to the degree of contact between the primary tumor and the vessels (portal vein (PV) or superior mesenteric vein (SMV), superior mesenteric artery (SMA), coeliac trunk, and common hepatic artery), PDAC is classified as resectable, borderline resectable, or locally advance unresectable. PDAC is considered resectable when the tumor is free of contact with the SMA, common hepatic artery, coeliac trunk, or contact of < 180° with SMV/PV without vessels’ contour irregularity; infiltration of SMA of ≥ 180 or the involvement or occlusion of SMV/PV is generally considered as locally advanced, unresectable disease. Then, intermediate vascular involvement identifies borderline resectable disease (**Table 1**).

**Table 1:** Criteria of resectability according to NCCN guidelines version 2.2021 (3).

Resectability Status	Venous	Arterial
Resectable	◼ No tumor contact with the superior mesenteric vein (SMV) or portal vein (PV) or ≤180° contact without vein contour irregularity.	◼ No arterial tumor contact (celiac axis [CA], superior mesenteric artery [SMA], or common hepatic artery [CHA]).
Borderline Resectable	◼ Solid tumor contact with the SMV or PV of >180° with contour irregularity of the vein or thrombosis of the vein but with suitable vessel proximal and distal to the site of involvement allowing for safe and complete resection and vein reconstruction. ◼ Solid tumor contact with the inferior vena cava (IVC).	Pancreatic head/uncinated process:
		◼ Solid tumor contact with CHA without extension to CA or hepatic artery bifurcation allowing for safe and complete resection and reconstruction. ◼ Solid tumor contact with the SMA of ≤180°. ◼ Solid tumor contact with variant arterial anatomy (ex: accessory right hepatic artery, replaced right hepatic artery, replaced CHA, and the origin of replaced or accessory artery) and the presence and degree of tumor contact should be noted if present, as it may affect surgical planning.
		Pancreatic body/tail: ◼ Solid tumor contact with the CA of ≤180°. ◼ Solid tumor contact with the CA of >180° without involvement of the aorta and with intact and uninvolved gastroduodenal artery thereby permitting a modified Appleby procedure (some panel members prefer these criteria to be in the locally advanced category).
Locally Advanced	◼ Unreconstructible SMV/PV due to tumor involvement or occlusion (can be due to tumor or bland thrombus).	Head/uncinated process:
		◼ Solid tumor contact with the SMA >180°. ◼ Solid tumor contact with the CA >180°. Pancreatic body/tail:
		◼ Solid tumor contact of >180°with the SMA or CA. ◼ Solid tumor contact with the CA and aortic involvement.

However, a careful multidisciplinary evaluation of those criteria is mandatory in each case in order to perform better patient selection; the multidisciplinary team should consist of a group of physicians from different specialties dedicated to PDAC, highly trained in this regard and working in a high-volume center. It should be assumed that patients with borderline resectable disease have a high probability of residual microscopic resection (R1 resection). For this reason, they should not be considered for upfront surgery and chemotherapy is the first option in the treatment strategy. On the other hand, patients with resectable disease at diagnosis are mainly receive upfront surgery as standard of care.

Nevertheless, despite curative resection, the rate of postoperative tumor recurrence is high and the majority of patients experience a disease relapse (4). On these bases, adjuvant chemotherapy should be offered to all patients who have undergone surgical treatment and maintain an acceptable general condition, regardless of pathological TNM stage, with the aim to improve the poor prognosis of these patients (3, 5). According to this concept, several phase III trials have been developed over the last decades in order to evaluate the more effective chemotherapy regimens, resulting in a radical change of management of resectable PDAC.

Based on this background, the aim of this narrative review is to provide an overview regarding the state of the art of adjuvant treatments in PDAC, alongside the emerging role of perioperative treatment. Lastly, we discuss the role of future perspectives in this field, such as biomarkers and new target therapies.

## Localized PDAC: What We Know in 2021 and the Current Treatment Scenario

After a suspicion of PDAC, cytological or pathologic diagnosis- usually made with fine-needle biopsy by endoscopic ultrasound guidance or computed tomography (CT)- is mandatory in cases of unresectable and borderline resectable disease (6). Then, an accurate preoperative CT staging and a multidisciplinary evaluation, focused on the assessment of distant metastasis and on the vessels’ involvement degree, is recommended in order to identify patients at risk of incomplete resection (R1 or R2 residual macroscopic disease). Those patients have a disappointing survival rate, similar to that of non-resected tumors in case of R2 resections. Additionally, a careful multidisciplinary evaluation might help to decrease the morbidity linked to a non-curative major surgery (5, 7–9).

According to international guidelines, patients with radiological resectable PDAC at diagnosis are candidates for surgery as standard of care, ideally performed in high-volume centers (3, 5). However, the multidisciplinary team should carefully evaluate patients with Ca 19-9 > 500 UI/ml, pain, or histological report of grade 3 tumor (so called “biological criteria of resectability”). In fact, those patients have higher risk of early relapse after surgery also in the case of radiological resectable tumors, underlining the systemic nature of PDAC. In those cases, a systemic treatment followed by curative surgery should be considered as a valid treatment strategy.

Thus, according to the location of the primary tumor, the surgical procedure can be a pancreatoduodenectomy (Whipple technique) in case of head and uncinate tumors and a distal pancreatectomy with en-bloc splenectomy in case of cancers in the body and tail. Regarding the definition of complete resection, the International Study Group of Pancreatic Surgery (ISGPS) recommends the following: R0 in case of negative resection margins; R1 in case of tumor cells within <1 mm from the margin, considering all seven margins (anterior, posterior, medial, superior mesenteric artery (SMA), pancreatic transection, bile duct, and enteric); and R2 in case of macroscopical residual disease (10). Additionally, surgery should include a standard lymphadenectomy with the removal of > 15 lymph nodes (11).

Currently, open surgery remains the standard of care for the treatment of PDAC, because laparoscopy has been shown to reduce peri-operative morbidity, but with no clear data about oncological results (12, 13). However, despite curative resection, the rate of postoperative tumor recurrence is high, and the majority of patients experience a disease relapse (4). Therefore, PDAC is considered a systemic disease from diagnosis even in cases of localized and resectable tumors. In these cases, a multimodal treatment strategy, such as surgery followed by an adjuvant chemotherapy, can offer more chances of survival (14). However, it is worth mentioning that up to 30% of patient do not receive adjuvant therapy because of the development of comorbidities, the worsening of performance status (PS), post-operative complications, and early recurrence.

Regarding adjuvant chemotherapies, several studies have been developed over the last decades. The European Study Group for Pancreatic cancer (ESPAC)-1 trial showed for the first time that a flourouracil-based adjuvant chemotherapy significantly increased survival compared to surgery alone (median overall survival (OS): 20.1 *versus* 15.5 months, respectively). Additionally, the trial showed a detrimental effect on survival by using an integrate approach with chemoradiotherapy if compared to chemotherapy (15).

Later, the CONKO-001 trial showed significant improvement in disease-free survival (DFS) by using gemcitabine-based adjuvant mono-chemotherapy *versus* observation in resectable PDAC (13.4 *versus* 6.9 months, respectively), whereas median OS was comparable between the gemcitabine and the control group (22.1 *versus* 20.2 months, respectively) (16).

The ESPAC-3 trial did a head-to-head comparison between the two regimens used in ESPAC-1 and in CONKO-001 trials (17). This trial showed no significant differences between the two treatment arms (median OS 23.0 months in the fluorouracil arm and 23.6 months in the gemcitabine arm), with a more acceptable safety profile in the gemcitabine arm (grade 3-4 toxicities: 7.5% *versus* 14% in the fluorouracil arm) (17). However, the ESPAC-3 trial underlined the concept that completing the adjuvant treatment for all six cycles planned, at appropriate dose intensity, has a major impact on survival, rather than an earlier beginning of chemotherapy within the 6-8 weeks after surgery. In fact, it showed that chemotherapy could be postponed for up to 12 weeks after surgery, allowing for a better recovery of patients (18).

More recently, two randomized clinical trials have deeply modified the standard of care for adjuvant chemotherapy for PDAC: ESPAC-4 and PRODIGE 24 trials (19, 20). In 2017, the ESPAC-4 trial showed that the combination of gemcitabine plus capecitabine (GEMCAP) was superior to gemcitabine alone with a significant but modest improvement in median OS (28.0 months in the experimental arm *versus* 25.5 months in the control arm, hazard ratio (HR): 0.82, p=0.032) (19). However, it is important to emphasize the absence of a significant difference in relapse-free survival (RFS) between the two arms, even though a trend in favor of the GEMCAP arm was reported (the 3- and 5-year survival rates were 23.8% and 18.6% with GEMCAP and 20.9% and 11.9% with monotherapy, respectively). The GEMCAP regimen was also associated with a poorer safety profile, with a higher percentage of grade 3-4 adverse events. Methodological limitations of this trial consist of the inclusion of patients with potentially poor prognosis, such as those with post-operative elevation of Ca19.9 serum level, and the absence of planned post-surgical radiological evaluation. Those factors suggest the presence of early metastatic disease in the study population, which might be the reason for the major efficacy of the combination regimen. Nevertheless, international guidelines consider the GEMCAP regimen as a valid option for adjuvant treatment (3, 5).

Then, the PRODIGE 24/CCTG PA.6 trial evaluated the role of a polichemotherapy based on modified fluorouracil/irinotecan/oxaliplatin regimen (mFOLFIRINOX) as adjuvant chemotherapy compared with gemcitabine alone (20). The trial reached its primary endpoint of increasing DFS in the majority of the subgroups (including R0 and R1 resections): after a median follow-up of 33.6 months, median DFS was 21.6 months in the mFOLFIRINOX arm *versus* 12.8 months in the gemcitabine arm (HR: 0.58; p<0.001). In addition, median OS was 54.4 months in the mFOLFIRINOX arm *versus* 35.0 months for gemcitabine arm (HR: 0.64; p=0.003); this was the best achievement in survival in this setting until now. As expected, grade 3-4 toxicities were significantly higher in the mFOLFIRINOX group (75.5% *versus* 51.1%), with higher rates of diarrhea, mucositis, fatigue, peripheral neuropathy, nausea, and vomiting. However, no grade 5 adverse events were reported in the experimental arm. Nevertheless, we should consider two aspects in the analysis of those results: first, only 66% of patients in the mFOLFIRINOX arm received all the planned cycles of chemotherapy compared to 79% in the control arm; second, the population of the PRODIGE 24 trial was very well selected (PS 0-1 according to ECOG, normal post-surgical radiological evaluation and Ca19.9 serum levels < 180 U/ml) with lower risk of early recurrence.

Additionally, the Italian phase III GIP-2 trial showed similar results in this setting, supporting the use of mFOLFIRINOX in the adjuvant setting (21). However, the trial was stopped earlier after the publication of the results of PRODIGE 24 trial, due to the low accrual.

In general, according to international guidelines (3, 5), mFOLFIRINOX is considered the best adjuvant strategy in very well selected and fit patients, with an optimal post-surgical recovery.

Finally, other trials were conducted with the aim to improve the outcomes in this setting. In particular, the APACT trial did not confirm the superiority in DFS of the combination of nab-paclitaxel plus gemcitabine when compared to gemcitabine alone (19.4 *versus* 18.8 months; HR: 0.88; p=0.1824) (22). Likely, the CONKO-005 trial, that evaluated the efficacy of adding erlotinib to gemcitabine, failed to demonstrate a benefit in DFS and OS in the adjuvant setting in the experimental arm (23).

Lastly, the potential impact of adjuvant radiation therapy to improve the outcome of patients with PDAC is still debated, due to the lack of definitive data evaluating modern radiotherapy doses and techniques. In fact, in the pivotal historical ESPAC-1 and EORT trials (that compared chemoradiotherapy with the observation after surgery alone), radiotherapy has been shown to not improve the survival outcomes in this setting, including in patients who have undergone R1 resection (15, 24).

However, we should consider that those first trials were conducted using suboptimal radiation regimens (such as split-course radiotherapy), without a standardization of doses and comparison groups.

On the other hand, two more recent studies using a national cancer registry database reported that chemordiotherapy was more effective than adjuvant chemotherapy alone, especially in node-positive status or R1 resection (25, 26). However, they were limited by potential inherent biases; therefore, their findings should be carefully interpreted. Thus, to date, the role of post-operative radiation in the modern era of new and more effective systemic therapies remains unanswered. It should be evaluated in phase III trials, at least in some categories of patients with higher risk of local recurrence.

In conclusion, mFOLFIRINOX is considered the best systemic treatment in the adjuvant setting in cases of selected and fit patients. On the other hand, gemcitabine-based monochemotherapy or GEMCAP regimen could be an option in the elderly and for patients with ECOG PS 2.

## PDAC Evolution: From Pancreatic Gland to Metastasis

Since the end of the 20th century, important advances in understanding pathological mechanisms beneath PDAC evolution have been made.

As for other human cancer types, a stepwise evolution model has been proposed for PDAC: tumor initiation, as a consequence of driver gene mutations; tumor progression, through clonal expansion and accumulation of new genetic alterations; and tumor dissemination, in which cancer cells reach, through the bloodstream, distant sites (27–29).

**Figure 1:**
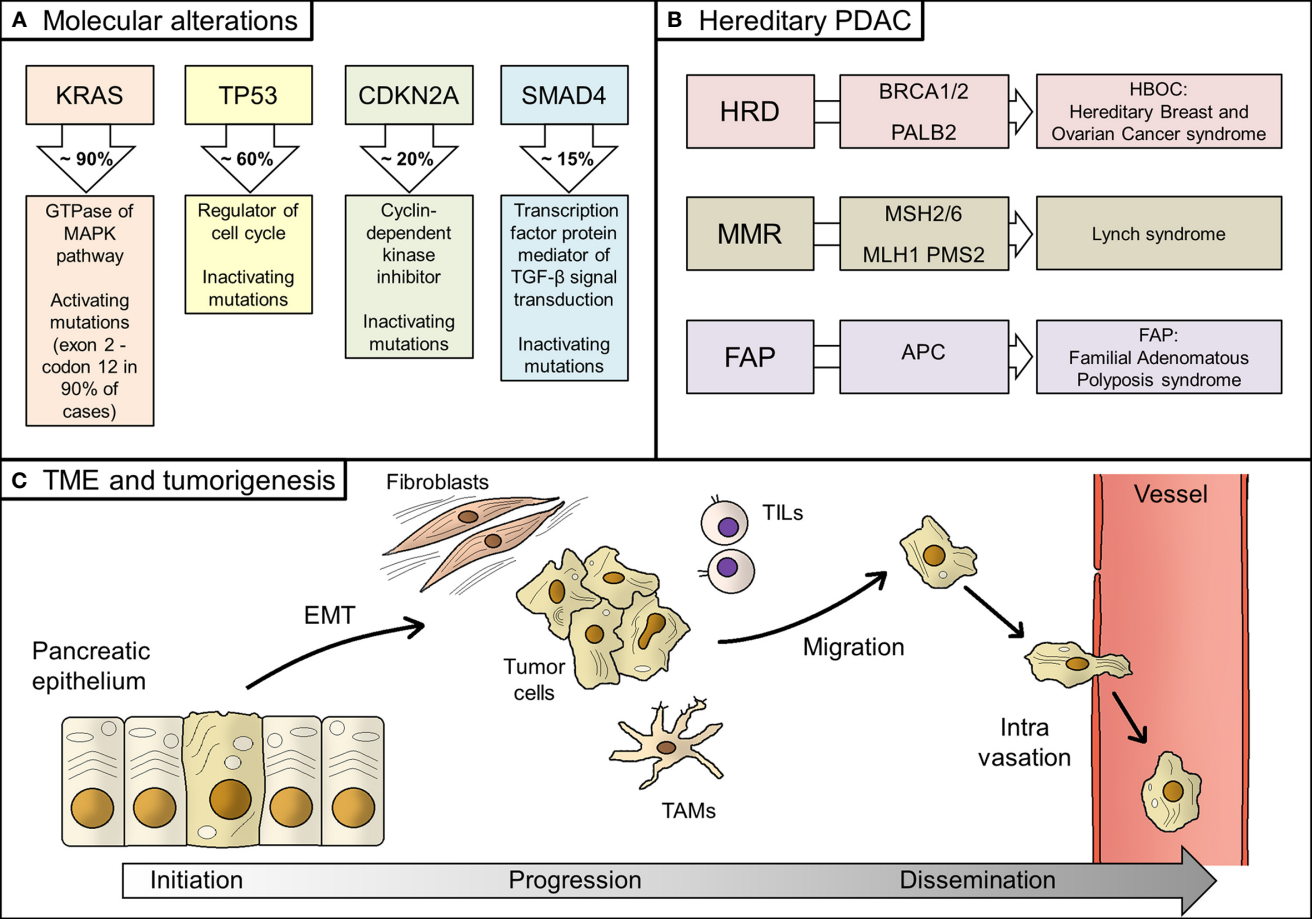
Biology of PDAC. **(A)** Main molecular alterations in PDAC; **(B)** Hereditary alterations, involved genes, and their relative syndromes; **(C)** Main steps of PDAC tumorigenesis, from tumor initiation to tumor progression and, finally, systemic dissemination. PDAC, pancreatic ductal adenocarcinoma; HRD, homologous recombination deficiency; TME, tumor microenvironment; EMT, epithelial-to-mesenchymal transition; TAMs, tumor-associated macrophages; TILs, tumor infiltrating lymphocytes; MMR, mismatch repair.

First genetic events in tumor initiation concern few loci (**Figure 1**). In particular, four genes (also known as the “Big 4”) are the most mutated in early pancreatic cancer, namely KRAS, TP53, CDKN2A, and SMAD4 (formerly known as DPC4), with their alterations detected for 94%, 64%, 21%, and 17% of all PDACs, respectively (30). KRAS mutations, which are localized in codon G12 in almost 90% of cases, are the earliest event in pancreatic tumorigenesis; they are activating mutations, unlike the other three genes (30, 31).

Transformation of normal pancreatic epithelium into malignant cells seems to cross in many cases through premalignant lesions, namely the pancreatic intraepithelial neoplasia (PanIN) and intraductal papillary mucinous neoplasm (IPMN) (32). In support of this hypothesis, genomic analysis of PanIN and IPMN showed that they share the same genetic alterations of PDAC, although with lower incidence (33).

Genetic alterations other than the abovementioned could pre-exist the tumor and be responsible for its onset. Hereditary PDAC account for 10% of all PDAC patients, even if a clear predisposition syndrome could be detected in no more than 20% of them (34). Hereditary alterations mainly affect BRCA1/2 genes and other homologous recombination genes, such as PALB2 (hereditary breast and ovarian cancer syndrome, HBOC), mismatch repair (MMR) genes (Lynch syndrome), and APC gene (Familial Adenomatous Polyposis syndrome, FAP) (35). BRCA1/2 and PALB2 alterations are the most frequent inherited mutations in PDAC patients, whilst MMR deficiency is rare, being recorded in less than 1% of patients (36, 37). However, genomic instability might underlie the onset of a significant percentage of PDACs (38, 39).

Epithelial-to-mesenchymal transition (EMT), consisting of the acquisition of migratory properties by epithelial cells, is a common feature in human cancer and it is also described in PDAC, linked to the generation of cancer stem cells, formation of metastasis, and resistance to therapy (40–42).

Then, the tumor microenvironment (TME) plays a fundamental role in PDAC genesis. TME is composed of stromal cells, extracellular matrix, immune cells, and blood vessels (43). It is not merely the physical and cellular support for the tumor growing, but its interactions with tumor cells are responsible for tumor behavior (i.e. promoting EMT), invasiveness, and metastasis (44, 45). In fact, stromal cells should be considered as dynamic elements, therefore representing potential therapeutic targets (46–48). Stromal fibroblasts promoted growth and metastasis in preclinical models of PDAC through production and secretion of soluble factors, whilst composition of immune infiltration has a critical role in tumor progression by regulating immune response against tumor cells (49–51). In detail, immune and inflammatory infiltration consists of several types of cells, from tumor-associated macrophages (TAMs) to bone marrow-derived cells (BMDCs), and from neutrophils to tumor-infiltrating T-cells (TILs) (52). Recent discoveries on antitumor immunity in PDAC have highlighted a peculiar immune microenvironment composition, which explains the evasion from immune surveillance by tumor cells (53). However, specific PDAC mutational signature, such as homologous recombinant deficiency- that results in higher frequency of somatic mutations- could enhance antitumor immunity and be a candidate for new immunotherapy drugs (54).

Tumor dissemination in the bloodstream is not an early event in the genetic evolution of PDAC since the metastatic ability is acquired only years after tumor initiation (55). However, on a clinical point of view, a considerable share of small PDACs (1-5 mm of diameter) are found with synchronous distant metastases, meaning that tumor clinically detectable masses have already accumulated several genetic alterations, thus leading to systemic disease. As support to those assumptions, circulating tumor cells (CTCs) have also been found in blood samples of PDAC patients in earlier stages (56–58). It should be noted that only a few CTCs have the ability to form metastases; however, despite the relative inefficiency of this process, the great amount of tumor cells released in bloodstream explains the high frequency of metastases (59, 60).

There are preferential sites for metastases from PDAC, such as liver, lungs, and peritoneum (61). Interestingly, in the last years several works have suggested the possibility that organs- in particular the liver- could be induced in accepting tumor cells through secreted factors released by primary tumor, such as inhibitor of metalloproteinases or exosomes (so called “pre-metastastic niches”) (62, 63). On the other hand, tumor cells could acquire specific characteristics for selective organotropism (64).

TME also plays an important role in the metastatic process. In particular, a similar extracellular matrix composition between primary and metastatic TME has been pointed out, even if metabolic genes in stromal cells are differentially expressed based on metastasis site, highlighting specific regulation in specific contexts (65, 66). Additionally, TME is not a static entity but changes over time in response to tumor behavior (67).

Lastly, on the genomic point of view, it seems that primary PDAC and synchronous/metachronous metastases share similar frequency in main tumor suppressor genes, even if a higher mutational load in cell cycle pathway genes has been observed in metastases (68). That observation supports the hypothesis that main genomic rearrangements involved in tumorigenesis of PDAC occur before bloodstream dissemination, which is indeed a late event in the natural history of this type of human cancer.

## New Directions in the Adjuvant Treatment of PDAC

### Better Stratification of Patients: Role of Biomarkers and Prognostic Factors

One of the biggest challenges in early-stage malignant tumors is to assess individual prognosis more accurately, specifically regarding risk of either local or distant relapse. Stratification of these patients is important to avoid unnecessary adjuvant treatment in those who will not experience disease recurrence but also to better tailor post-operative treatment – i.e. number and type of drugs administered or treatment duration – in those who have a high probability of micro-metastases.

Radiological exams currently used in clinical practice are unable to detect micro-metastatic disease; this is why biomarkers, especially those correlating with metastatic burden, have been intensively studied in early PDAC patients in the last decades in order to ideally separate those patients who are at high risk of distant recurrence from those who are not (69).

Among serum biomarkers detectable on blood samples, Ca 19-9 is certainly the most diffused and studied. In fact, elevated levels of Ca19-9 have been associated with poor survival in early stage PDAC patients (70). However, its sensitivity and specificity as a single agent does not allow its use in early detection of PDAC, explaining the clinical need of serological partners to be tested with it (71–73). For example, a recent study pointed out the possibility to predict disease recurrence in PDAC patients through the combination of Ca19-9 and serum metabolomes (74).

**Table 2** summarizes novel potential biomarkers and prognostic factors in localized PDAC patients and their potential influence on choosing the best curative approach. Among those, CTCs and circulating tumor DNA (ctDNA) are really promising. CTCs have been detected in early stage PDAC patients. In particular, in clinically and radiologically localized PDAC a cut-off of 3 CTCs per vial (4 ml) was proposed in the literature to discriminate between patients with or without distant micrometastases (75). CTCs are undoubtedly useful in risk stratification, being a candidate for clinical implementation in the near future (76–78).

**Table 2:** Potential biomarkers and prognostic factors in localized PDAC patients and their potential influence on choosing the best curative approach.

Biomarker/prognostic factor	Optimal timing for use	Influence on curative approach(es)
CTCs*	◼ Detection before surgery indicates a high probability of distant (micro)metastases.	◼ Neoadjuvant chemotherapy should be considered.
	◼ Detection after surgery indicates a high probability of distant (micro)metastases and/or residual disease.	◼ “Adjuvant” chemotherapy should be strengthened.
ctDNA**	◼ Detection before surgery indicates a high probability of distant (micro)metastases.	◼ Neoadjuvant chemotherapy should be considered.
	◼ Detection after surgery indicates a high probability of distant (micro)metastases and/or residual disease.	◼ “Adjuvant” chemotherapy should be strengthened.
NLR and PLR §	◼ High value after surgery may suggest poor prognosis in frail patients.	◼ Adjuvant chemotherapy should be avoided.

*Circulating tumor cells, **Circulating tumor DNA, §Neutrophil-to-lymphocyte ratio (NLR) and platelet-to-lymphocyte ratio.

Regarding ctDNA- which is a hot topic in cancer research worldwide- its role in early stage PDAC has been more finely shaped in very recent years (79). The most important study about ctDNA in early stage PDAC patients has been conducted on 112 subjects suitable for radical resection of primary tumor: pre- and post-operative detection of KRAS mutations in ctDNA was correlated with poor RFS and OS in PDAC patients, also including those who received adjuvant therapy (80). The trial suggested that patients with detectable levels of ctDNA should be treated more aggressively after resection. In order to increase the role of ctDNA as a predictive biomarker, Hussung et al. demonstrated that integration of Ca19-9 and KRAS mutant ctDNA performed better than individual markers for both RFS and OS in PDAC patients undergoing adjuvant treatment (81). Extracellular vesicles are also promising biomarkers for early stage PDAC patients, but optimization of analytical processes is needed for practical use (82).

Beyond tumor biomarkers and moving from the evidence in the metastatic setting, systemic inflammation markers have also been studied in radically resected PDAC patients, such as neutrophil-to-lymphocyte ratio (NLR) and platelet-to-lymphocyte ratio (PLR) (83–85). Recently, a systemic immune-inflammation index, which is the ratio between platelets × neutrophils and lymphocytes, has been proposed as a new prognostic score, predicting poor survival with more accuracy than NLR and PLR (86).

Then, Kim et al. structured a nomogram for early recurrence after pancreatectomy in localized PDAC in order to help clinicians in predicting recurrence risk. The nomogram included some of the abovementioned prognostic (namely, Ca 19-9, NLR, and PLR) and pathological factors (such as tumor size and grade of differentiation) (87). Eventually, in the future, the integration of other biomarkers in some nomograms - such as CTCs and ctDNA –will certainly enhance their predictiveness. Additionally, their use in adjuvant clinical trials should be encouraged for tailoring therapy based on the risk of disseminated microscopic disease.

### New Potential Targets in the PDAC Complex Scenario

PDAC is a very complex and heterogeneous disease at the molecular and clinical level. In fact, in the adjuvant context, only a few examples regarding molecular predictive biomarkers exist and no targeted agents are currently used in clinical practice in this setting.

Martinelli P et al. used the large ESPAC-3 trial cohort to classify patients according to the level expression of GATA6 transcription factor, a putative marker of Collisson and Moffitt “Classical” subtype (17, 88–90). They clearly showed that individuals with high GATA6 expression (what we consider as the “classical” type) received the greatest benefit from adjuvant 5-Fluorouracil administration, whereas patients with low-GATA6 did not benefit by using this type of chemotherapy at all. To note, no survival differences based on GATA6 expression were found in the gemcitabine-based adjuvant arm. The hypothesis, also supported by the recent COMPASS trial in advanced disease setting, is that the classical subtype could be more sensitive to fluoropyrimidine, even in the adjuvant context, making GATA6 an “ideal” (and relatively simple) marker to assess in order to choose the better adjuvant strategy (91).

Buchler’s group, again using the data from ESPAC-3 trial (17), showed the potential utility of another marker, hENT1 (human equilibrative nucleoside transporter 1), to predict benefit by using a gemcitabine-based adjuvant therapy (92). In details, hENT1 permits the bidirectional passage into cancer cells of pyrimidine nucleosides (such as gemcitabine, 5-Fluorouracil, and capecitabine), which suggests that higher levels of this transporter could correlate with increased intracellular accumulation of chemotherapy agents, thus causing cancer cell death. Indeed, this retrospective analysis showed that patients who received a gemcitabine-based chemotherapy had a median OS of 26.2 months in case of high hENT1 expression level; on the other hand, patients with low hENT1 levels showed a median OS of 17.1 months after gemcitabine. Nevertheless, there was no difference in hENT1 expression levels in the 5-Fluorouracil-based chemotherapy arm. These preliminary data were also recently confirmed by a Korean study, making hENT1 a possible predictive biomarker for clinical benefit by using a gemcitabine-based adjuvant regimen (93). To explain these results, it is interesting to note that hENT1 has been reported to be the most efficient transporter for gemcitabine but not for other pyrimidine nucleosides (94). Additionally, *in vitro* studies have shown that hENT1 loss could be responsible for resistance to gemcitabine in gastrointestinal human cancer cell lines (95).

More recently, Nicolle R et al. expanded our knowledge about molecular stratification in the adjuvant setting, identifying a molecular signature (the so-called “GemPred” signature) able to predict benefit from adjuvant administration of gemcitabine. Less than 20% of the retrospectively tested patients (~ 430 from different cohorts) were GemPred signature positive, all with “classic” transcriptomic features (96). Interestingly, the median DFS in patients with GemPred positive signature was longer than those with GemPred negative signature (42.5 *versus* 13.4 months); similar results were obtained for the median OS (91.3 *versus* 31.7 months). What kind of molecular intersections there are between GemPred and classic signatures has not been defined yet. However, it is a matter of fact that all patients with GemPred positive signature also had the classic PDAC subtype, whose sensitivity to 5-Fluorouracil was previously shown by Martinelli P et al. (87). Therefore, a better comprehension of the relationship between classic signature and this novel GemPred signature is highly desirable, also in the light of novel single cell data.

A major barrier to precision medicine in PDACs is the inter- and, especially, intra-tumor heterogeneity. Recent data have clearly shown that in a single tumor- defined as classical or basal-like at the bulk level- there is a transcriptional continuum at single cell level between classical and basal-like transcriptional programs (97). Thus, some cells are in a “classical-like state” and others in a “basal-like state”, possibly due to different microenvironmental interactions and spatial location within the different tumor regions. This notion complicates the picture further, representing a possible barrier to cytotoxic and/or targeted treatments directed to one specific “bulk” subtype.

#### Targeted Therapies in the Adjuvant Treatment for PDAC: Hope or Chimera?

Another crucial question in the adjuvant setting for PDAC is the following: beyond classical chemotherapy agents (see *section 2 for additional details*), what specific molecular targets could we imagine in the adjuvant setting? Necessarily we should look at genomic characterizations and at metastatic disease setting.

The first attempt to target metastatic PDAC with a molecular agent was published in 2007, with the combination of gemcitabine and the anti-epithelial growth factor receptor (EGFR)/tyrosine kinases inhibitor (TKI) Erlotinib, based on the observed overexpression of this receptor in tissue from PDAC (98, 99). Although the phase III trial met its primary endpoint with a statistically significant improvement in OS with the combination (6.24 *versus* 5.91 months, respectively), this survival gain was clinically irrelevant. Thus, to date, Erlotinib is not used in the clinical practice for metastatic PDAC. Erlotinib was also tested in the adjuvant setting in combination with gemcitabine and compared to gemcitabine alone (RTOG 0848 trial (100). Preliminary results were negative, showing a lack of survival benefit from the addition of erlotinib to standard chemotherapy. It must be underlined that in both cases (metastatic and adjuvant setting) the study population was not selected by EGFR expression and/or EGFR gene amplification, which could explain – at least in part – the disappointing results.

A recently published retrospective analysis of tumor specimens from CONKO-005 trial has suggested that a specific genetic signature - SMAD4 gene alterations with low MAPK9 expression - could be responsible for erlotinib efficacy in the adjuvant setting, even if these results need to be prospectively validated (101).

**Figure 2:**
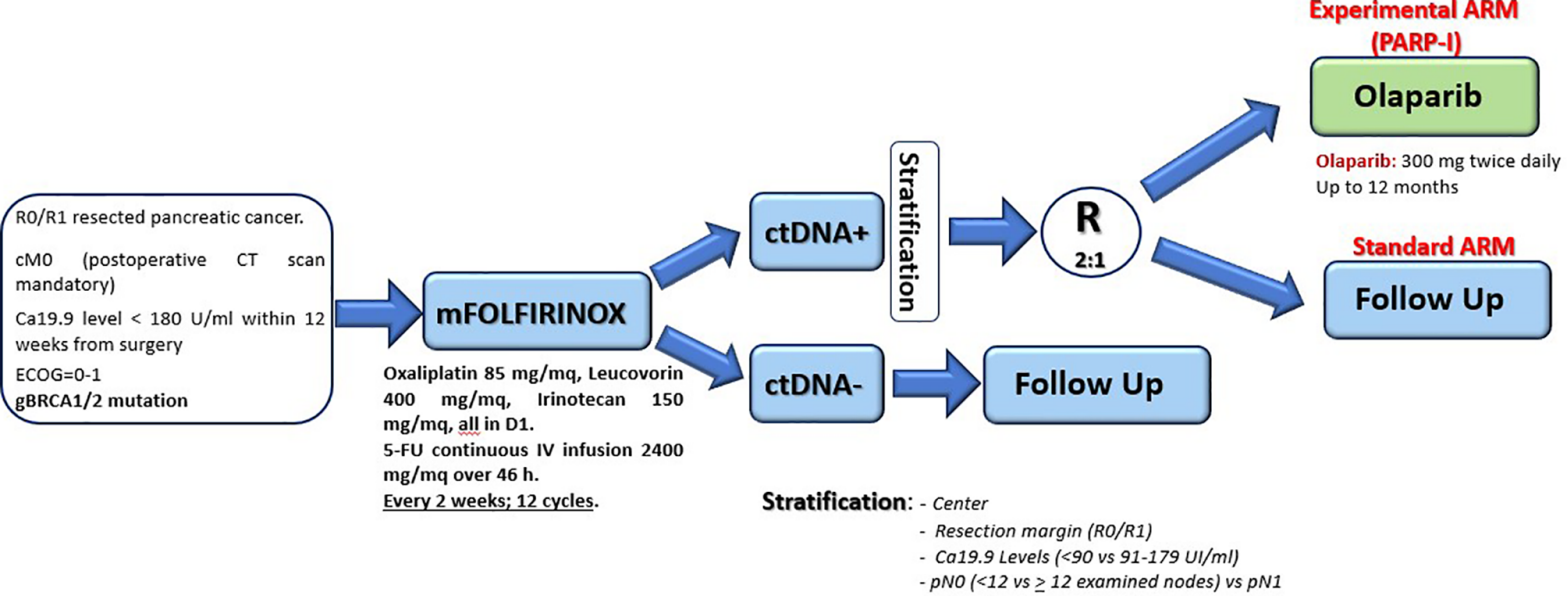
Hypothetic adjuvant study design dedicated to germline BRCA1/2 mutated PDAC patients.

In 2015, the consortium led by Biankin and Grimmond identified a small percentage of PDAC (< 15%) with high genomic instability due to serious defects in DNA integrity maintenance (creating the so called “BRCA signature”) (102). These patients showed alterations in genes like BRCA1, BRCA2, and PALB2, and the authors could demonstrate a clear clinical usefulness of a platinum-based chemotherapy, at least in two subjects, also assuming a possible role of PARP inhibitors in this context. Based on those preliminary results, the phase III POLO trial evaluated the efficacy of maintenance therapy with Olaparib (a PARP inhibitor) in germline BRCA1/2 mutated metastatic PDAC patients. The trial showed doubled median PFS (from 3.8 to 7.4 months) after an induction first-line therapy platinum-based (103). Although those results were promising, data regarding a possible adjuvant use of Olaparib in radically resected patients are not yet available. Nevertheless, a hypothetic study design as maintenance strategy (up to one year) in ctDNA positive germline BRCA1/2 mutated patients after mFOLFIRINOX standard adjuvant therapy is reported in **Figure 2**; **Table 3** shows the ongoing trials in this field.

**Table 3:** Selected major ongoing studies investigating new perioperative/adjuvant approaches.

Study	Phase	*N* of patients	Setting	Experimental arm	Status
NeoPancONE	II	84*	Perioperative	FOLFIRINOX x 6 periop. (GATA-6 expression)	Recruiting
NCT04472910 (104)					
PROJECTION	Observational	200*	Neoadjuvant	ctDNA detectable *vs* absent in preoperative	Recruiting
NCT04246203 (105)					
NCT01072981	III	722	Adjuvant	Gemcitabine or 5FU chemoradiation +/-Algenpantucel-L	Completed
(HyperAcute-Pancreas Immunotherapy) (106)					
DECIST	I	43*	Adjuvant	Standard chemo + autologous DC** vaccine	Recruiting
NCT04157127 (107)					
NCT04117087	I	30*	Adjuvant	KRAS peptide vaccine§ + nivolumab ipilimumab	Recruiting
(Pooled Mutant KRAS-Targeted Long Peptide Vaccine) (108)					
NCT00733746 (109)	II	123	Perioperative	Erlotinib + gemcitabine periop.	Completed

*estimated, **Dendritic Cells, §Hiltonol^®^ (Poly-ICLC).

A very small percentage of PDAC shows high microsatellite instability (MSI-H), a molecular feature associated with high response to immune checkpoint inhibitors (ICIs) in advanced disease setting across multiple cancer types (110, 111). However, it is a matter of fact that the objective response rate (ORR) of PDAC to ICIs was lower than that observed in other types of MSI-H cancers (112). However, an adjuvant approach with ICIs in MSI-H patients with ctDNA might be worth investigation in the adjuvant setting in the future.

Nevertheless, with the exception of rare MSI-H patients, PDAC is considered a tumor resistant to ICIs, due to a highly immune-suppressive microenvironment, dominated by extracellular matrix proteins and different cancer associated fibroblast subtypes as well as other immune cell types (113). A very recent report from the NCT02451982 phase I/II Trial is evaluating the combination of GVAX (tumor cell vaccine) plus Nivolumab (anti-PD1) and Urelumab (CD137 agonist). Unfortunately, the results of the adjuvant phase of the trial are not available yet (see *section 4.3 for additional details regarding the results in the neoadjuvant setting*) (114).

Another interesting strategy in the adjuvant setting is to add chloroquine to gemcitabine, thus targeting autophagy, a resistance mechanism to chemotherapy, which has a role in PDAC maintenance, possibly also in a micro-metastatic state (115). In this regard, only the results of the phase II trial in a metastatic setting are available to date (116). The trial did not show any survival benefit for the chloroquine arm; however, a significant improvement in ORR was reported. Based on that consideration, the adding of chloroquine could make even more sense in a pre-operative setting for borderline resectable and/or locally advanced PDAC, where good tumor response could lead to radical surgical resection. However, further prospective evaluations are needed in order to explore this hypothesis.

In conclusion, target therapies are not considered the standard of care in the adjuvant setting for PDAC and they are not used outside clinical trials.

### How to Improve the Outcomes for Resectable PDAC: The Role of Neoadjuvant and Perioperative Treatments

The role of neoadjuvant treatment in PDAC is still controversial to date, although several trials and retrospective studies have been conducted in this setting and the general trend is to encourage this approach in the light of the systemic behavior of this malignancy. However, evidence available is still not univocal, as different clinical entities are currently investigated together for primary chemotherapy: locally advanced unresectable, borderline resectable, and resectable tumors (see **Table 1**). All these entities account for 50-60% of the whole newly diagnosed PDAC, but less than half are borderline or upfront resectable tumors.

For borderline/locally advanced tumors, an “induction” treatment should be conceptualized rather than a real “neoadjuvant”, even if no robust data have been reported. Preoperative treatment is able to achieve a radical resection in approximately 30% of the cases initially deemed unresectable, while almost 70% of the resectable cases regularly undergo surgery after a neoadjuvant therapy. However, as already discussed in the previous sections, a high percentage of resected patients are bound to relapse despite the best surgical and systemic approach currently available, and not all patients receive chemotherapy after surgery. Potential key points to improve survival outcomes in “curable” settings are the possibility to intensify and individualize pre- and postoperative treatment and the possibility to “adapt” adjuvant regimens in light of neoadjuvant response rate and/or biomarkers expression. This last point is getting more topical the more neoadjuvant treatment is growing in importance in the therapeutic algorithm.

Is it possible to outline a possible perioperative strategy according to specific clinical and biological markers? As a matter of fact, a major reason for treatment failure both in resectable and in locally advanced PDAC is the clinical and biological heterogeneity of different treated tumors, as well as the strong systemic “vocation” of this malignancy since the initial stages. Current neoadjuvant and adjuvant schedules are not able to tackle these issues in most cases.

During the last years, intensified regimens, such as gemcitabine plus nab-paclitaxel and FOLFIRINOX, have been proposed in order to improve resection rate and survival in the neoadjuvant setting.

The use of FOLFIRINOX, established as a standard in adjuvant and metastatic settings, seems suitable and promising according to meta-analytic data, while no prospective phase III data are available in this setting to date (117). In 2019, a meta-analysis of 24 small retrospective and phase I-II prospective studies highlighted the role of this combination in borderline resectable PDAC (1802 patients). The analysis showed a pooled resection rate of 67.8% (95% Confidence Interval (CI): 60.1%-74.6%) and R0-resection rate of 83.9% (95% CI: 76.8% - 89.1%) among all resected patients for the 13/24 studies reporting data about resection margins (117). The median OS ranged from 11.0 to 34.2 months across the studies (to note: lower than phase III PRODIGE-24 with adjuvant FOLFIRINOX). These data are consistent with previously reported meta-analysis (118). Besides the absence of a dedicated randomized controlled trial, the biggest limitation for the use of FOLFIRINOX is the toxicity, with grade 3-4 neutropenia, diarrhea, and fatigue usually reported as the most common adverse events.

With regards to gemcitabine and nab-paclitaxel combination, the Italian phase II GAP trial is the only randomized study comparing this combination *versus* gemcitabine alone (119). In locally advanced tumors, gemcitabine and nab-paclitaxel performed better in terms of distant relapses, also positively affecting PFS and OS, compared to gemcitabine monotherapy. The combination reduced rate of patients who progress after 3 cycles of induction chemotherapy by 20%.

In 2020, the LAPACT phase II single arm trial confirmed the role nab-paclitaxel and gemcitabine (6 cycles) as induction treatment in patients with locally advanced PDAC, with promising PFS (10.9 months, 90% CI; 9.3-11.6) and OS (18.8 months, 90%CI; 15.0- 24.0) and a good tolerability (consistently with data from the metastatic setting) (120). The data reported a better survival compared to historical reference trials. The response rate was 33.6% (90% CI: 26.6 - 41.5), establishing a good activity of this combo for locally advanced PDAC (121, 122).

However, currently no prospective head-to-head comparison between these two schedules (FOLFIRINOX *versus* gemcitabine and nab-paclitaxel) in the “induction” setting for resectable disease is available, whereas the majority of the evidence is retrospective or related to the locally advanced disease (123). The randomized American phase II trial SWOG S1505 compared perioperative treatment using mFOLFIRINOX *versus* gemcitabine and nab-paclitaxel in patients with resectable PDAC (104). The trial enrolled 147 patients and preliminary results, presented at the 2020 ASCO meeting, showed no significant differences in terms of OS (primary endpoint) between the two combinations (22.4 *versus* 23.6 months), with similar resection rates (77% *vs* 73%) (122). The SWOG S1505 and the phase III PREOPANC-1 are currently the most recent and robust evidence supporting the feasibility of perioperative treatment respectively in resectable and borderline resectable PDAC, assuming the use of the same regimens in the postoperative setting (FOLFIRINOX/gemcitabine and nab-paclitaxel versus gemcitabine, respectively) (104, 124). Noteworthy, PREOPANC-1 was not able to demonstrate a significant benefit in OS with the use of preoperative chemoradiotherapy compared to upfront surgery followed by adjuvant gemcitabine (35.2 *versus* 19.8 months; p = 0.029), while a significant higher R0-resection rate was reached (71% *versus* 40%, p < 0.001) (124).

A further contribution to the evaluation of neoadjuvant chemotherapy in resectable PDAC comes from the Italian phase II PACT-15 trial published in 2018 (106). In this randomized open-label study (93 patients), authors investigated an intensified perioperative approach with PEXG (cisplatin, epirubicin, gemcitabine, and capecitabine) in comparison with the same schedule as adjuvant treatment or a standard adjuvant gemcitabine. In the perioperative arm, 66% of patients were event-free at 1-year (primary endpoint) *versus* 23% and 50% in the other arms, respectively. Although of phase II design, this study provided further evidence of the feasibility and promising efficacy of neoadjuvant chemotherapy in resectable tumors, and was one of the few direct comparisons with adjuvant treatment.

In summary, according to these trials (SWOG S1505, PREOPANC-1, and PACT-15), the trend should go towards the repurpose of the same regimen, used as neoadjuvant, in the postoperative setting, while both FOLFIRINOX and gemcitabine/nab-paclitaxel should be considered feasible for preoperative treatment in resectable and/or locally advanced tumors (104, 106, 124).

How much adjuvant treatment adds after preoperative treatment and whether it should be selected according to clinical features are interesting points still to be clarified. Some data are available with the use of lymph node ratio (LNR) as a prognostic marker after neoadjuvant treatment followed by surgery, in order to stratify the efficacy of adjuvant therapy according to this factor. From an American registry database, including patients with PDAC who underwent resection following neoadjuvant chemotherapy until 2008, clinicopathologic factors have been retrospectively analyzed (107). Among the 14% of patients who also received postoperative therapy, the treatment was associated with better survival (72 *versus* 33 months, p = 0.008) in those with an LNR < 0.15, as confirmed by multivariate analysis. The addition of postoperative chemotherapy after neoadjuvant resulted in improved outcomes (reduced risk of death of 51%, p = 0.02, and longer time-to-recurrence) in patients with low LNR. However, this study reported a lack of benefit by the addition of adjuvant treatment in patients with severe lymph node involvement, contradicting other retrospective studies that showed - by contrast - a survival benefit from postoperative chemotherapy especially in patients with node-positive status (105, 107, 108). It is not clear whether the pathological node status plays a stronger prognostic role itself rather than a positive predictive meaning for adjuvant treatment.

No data are available about the role of tumor regression grade (rarely applied for pancreatic cancer) and R0-resection rate in the choice of prosecution and type of adjuvant schedule after neoadjuvant treatment.

All things considered, the open questions for future research in the perioperative context could be the following: the role of adjuvant treatment in pN0 patients, the role of neoadjuvant treatment in upfront resectable tumors (versus the exclusive adjuvant approach), the prosecution of adjuvant treatment in poor responsive patients treated with preoperative chemotherapy (such as switch adjuvant strategy or switch therapy in the non-responders), and the choice of what first line treatment should be used at disease relapse considering all the therapies used in the perioperative approach. In fact, in this challenging scenario, the role of adjuvant chemotherapy after upfront surgery might evolve, since the patients who will undergo immediate pancreatic resection are those in the very early stages who do not need neoadjuvant/perioperative approaches. Additionally, the biologic features and changes in patients who underwent induction chemotherapy followed by surgery should also be considered. About this last point, interesting data are expected from the phase II NeoPancONE trial, which is investigating the molecular features of resectable PDAC that underwent radical surgery after neoadjuvant FOLFIRINOX (109). One of the main aims of this study will be to analyze a potential biomarker already identified in the COMPASS trial, GATA-6, in the perioperative strategy, with the aim to stratify tumor types and responses to treatment (90). This may offer a chance to better select “different regimens for different patients”. This study will be the first able to correlate a potential biomarker to a neoadjuvant chemotherapy.

Additionally, considering the promising prognostic role of ctDNA, its detection after surgery could become a biomarker of response to neoadjuvant chemotherapy and help the clinicians to optimize the post-operative approach in case of poor responsive patients (80, 125).

The landscape of targeted therapy and immunotherapy for PDAC is still disappointing, mainly because of the uncomplete knowledge of the complex mechanisms underlying this malignancy and its intricate relations with the tumor microenvironment (as already mentioned in the previous section). With regards to immunotherapy and vaccine research in the neoadjuvant setting, a multi-institutional phase III study has been conducted using algenpantucel-L (a cancer vaccine comprised of irradiated allogeneic transfected pancreatic cancer cells) in addition to adjuvant chemotherapy or chemoradiotherapy (gemcitabine or 5-fluorouracil), based on the results of the phase II in 2013 (126, 127). The transfected cells are able to synthetize a murine enzyme, which is responsible for the production of a cell-surface protein (α-1,3-galactosyl (αGal) carbohydrate) not expressed in humans. The binding of preexisting natural human antibodies (naturally produced against the same proteins of the gut flora, accounting for 1% of all circulating immunoglobulins) results in the activation of antibody-dependent cell-mediated cytotoxicity toward allograft cells and endogenous pancreatic cancer cells. This process, through the so-called “epitope spreading”, expands the immune response against other tumor-associated antigens expressed by both injected cells and native cancer cells. The vaccine “drives” a natural preexisting immune weapon against pancreatic cancer, boosting chemotherapy to obtain a response against pancreatic cancer cells, normally resistant to the immune system. In the phase II trial, 70 resected patients have been treated with a 12-month DFS rate of 62% and a 12-month OS of 86%, describing site pain and induration as the most common adverse event. Further definitive efficacy results are expected.

Other phase I/II trials involving vaccine and immunotherapy in the adjuvant setting will provide a further attempt to turn “cold” pancreatic cancer into “hot” immune-sensitive disease (128–130). For additional details regarding ongoing trials in this setting, see **Table 3**.

## Conclusions

PDAC treatment has hugely improved in recent decades. In fact, even if the use of gemcitabine has been the better therapeutic chance for those patients for a long time, both in adjuvant and in metastatic settings, a lot of new drugs and strategies are appearing in therapeutic armamentarium today. However, PDAC remains a big challenge in the oncological scenario. In fact, even in cases of curative surgery for resectable disease, the rate of recurrence is high, suggesting an early systemic diffusion of cancer cells. A multidisciplinary evaluation of PDAC patients in high volume centers could help to improve the outcomes for those patients, by creating a tailored therapeutic strategy for each patient. According to international guidelines (3, 5), to date, adjuvant chemotherapy based on mFOLFIRINOX or gemcitabine is the recommended treatment in patients with resectable PDAC after curative surgery. However, many changes are ongoing in the current treatment scenario. In particular, use of perioperative and neoadjuvant treatment, even for resectable and borderline resectable PDAC, might allow the adjuvant chemotherapy after upfront surgery to play a marginal role in the future. Additionally, a better understanding of the molecular mechanism of PDAC as well as the research about prognostic and/or predictive biomarkers is urgently needed in order to better select patients who can benefit from different and/or personalized approaches and to design future prospective clinical trials regarding targeted therapies also in this field.

## Author Contributions

Conceptualization: APa, EG, and APe. Topic: APa, EG, and APe. resources: APa and EG. writing—original draft preparation: APa, EG, and APe. Writing of particular sections: all authors. Writing—review and editing: all authors. Supervision, APe, BD, and FV. All authors contributed to the article and approved the submitted version.

## Conflict of Interest

EG had personal fees from Novartis. GT received a travel grant from Servier, Italfarmaco, advisory board: Eli-Lilly. BD received personal fees from Ipsen, Eisai, Eli Lilly, Astra Zeneca, Sanofi, MSD, Bayer, Roche, and Amgen. FV: Scientific consultancy: Servier, Lilly, MSD and BMS; honoraria for speaking: Roche, Bayer, Servier, Lilly, Astellas, MSD and BMS. APe had personal fees with Eli-Lilly, Servier, and MSD. No fees are connected with the submitted paper.

The remaining authors declare that the research was conducted in the absence of any commercial or financial relationships that could be construed as a potential conflict of interest.

## Publisher’s Note

All claims expressed in this article are solely those of the authors and do not necessarily represent those of their affiliated organizations, or those of the publisher, the editors and the reviewers. Any product that may be evaluated in this article, or claim that may be made by its manufacturer, is not guaranteed or endorsed by the publisher.

## References

[1] Rahib L Smith BD Aizenberg R Rosenzweig AB Fleshman JM Matrisian LM. Projecting Cancer Incidence and Deaths to 2030: The Unexpected Burden of Thyroid, Liver, and Pancreas Cancers in the United States. Cancer Res (2014) 74:2913–21. 10.1158/0008-5472.CAN-14-0155 24840647

[2] Varadhachary GR Tamm EP Abbruzzese JL Xiong HQ Crane CH Wang H. Borderline Resectable Pancreatic Cancer: Definitions, Management, and Role of Preoperative Therapy. Ann Surg Oncol (2006) 13(8):1035–46. 10.1245/ASO.2006.08.011 16865597

[3] Ducreux M Cuhna S Caramella C Hollebecque A Burtin P Goéré D. Cancer of the Pancreas: ESMO Clinical Practice Guidelines for Diagnosis, Treatment and Follow-Up. Ann Oncol (2005) 26:56–8. 10.1093/annonc/mdv295 26314780

[4] Katz MH Wang H Fleming JB Sun CC Hwang RF Wolff RA. Long-Term Survival After Multidisciplinary Managenement of Resected Pancreatic Adenocarcinoma. Ann Surg Oncol (2009) 16:836–47. 10.1245/s10434-008-0295-2 PMC3066077 19194760

[5] National Comprehensive Cancer Networks. NCCN Guidelines Version 2.2021 (2021). Available at: http://www.nccn.org (Accessed Accesed February 25, 2021).

[6] Asbun HJ Conlon K Fernandez-Cruz L Friss H Shrikhande SV Adham M. International Study Group of Pancreatic Surgery. When to Perform a Pancreatoduodenectomy in the Absence of Positive Histology? A Consensus Statement by the International Study Group of Pancreatic Surgery. Surgery (2014) 155:887–92. 10.1016/j.surg.2013.12.032 24661765

[7] Tamm EP Balachandran A Bhosale PR Katz MH Fleming JB Lee JH. Imaging of Pancreatic Adenocarcinoma: Update on Staging/Resectability. Radiol Clin North Am (2012) 50:407–28. 10.1016/j.rcl.2012.03.008 22560689

[8] Hernandez J Mullinax J Clark W Toomey P Villadolid D Morton C. Survival After Pancreaticoduodenectomy is Not Improved by Extending Resections to Achieve Negative Margins. Ann Surg (2009) 250:76–80. 10.1002/bjs.11115 19561479

[9] Bilimoria KY Talamonti MS Sener SF Bilimoria MM Stewart AK Winchester DP. Effect of Hospital Volume on Margin Status After Pancreaticoduodenectomy for Cancer. J Am Coll Surg (2008) 207:510–9. 10.1016/j.jamcollsurg.2008.04.033 18926452

[10] Bockhorn M Uzunoglu FG Adham M Imrie C Milicevic M SandBerg AA. Borderline Resectable Pancreaticcancer: A Consensus Statement by the International Study Group of PancreaticSurgery (ISGPS). Surgery (2014) 155:977–88. 10.1016/j.surg.2014.02.001 24856119

[11] Tol JA Gouma DJ Bassi C Dervenis C Montorsi M Adham M. Definition of a Standard Lymphadenectomy Insurgery for Pancreatic Ductal Adenocarcinoma: A Consensus Statement by Theinternational Study Group on Pancreatic Surgery (ISGPS). Surgery (2014) 156:591–600. 10.1016/j.surg.2014.06.016 25061003 PMC7120678

[12] Kooby DA Hawkins WG Schmidt CM Weber SM Bentrem DJ Gillespie TW. A Multicenter Analysis of Distalpancreatectomy for Adenocarcinoma: Is Laparoscopic Resection Appropriate? J Am Coll Surg (2010) 210:779–785.24. 10.1016/j.jamcollsurg.2009.12.033 20421049

[13] Ricci C Casadei R Taffurelli G Toscano F Pacilio CA Bogoni S. Laparoscopic Versus Open Distalpancreatectomy for Ductal Adenocarcinoma: A Systematic Review and Meta-Analysis. J Gastrointest Surg (2015) 19:770–81. 10.1007/s11605-014-2721-z 25560180

[14] Strobel O NJ Jäger D Markus W Büchler MW. Optimizing the Outcomes of Pancreatic Cancer Surgery. Nat Rev Clin Oncol (2018) 16:11–26. 10.1038/s41571-018-0112-1 30341417

[15] Neoptolemos JP Dunn JA Stocken DD Almond J Link K Beger H. Adjuvant Chemoradiotherapy and Chemotherapy in Resectable Pancreatic Cancer: A Randomised Controlled Trial. Lancet (2001) 358:1576–85. 10.1016/s0140-6736(01)06651-x 11716884

[16] Oettle H Post S Neuhaus P Gellert K Langrehr J Ridwelski K. Adjuvant Chemotherapy With Gemcitabine *vs* Observation in Patients Undergoing Curative-Intent Resection of Pancreatic Cancer: Arandomized Controlled Trial. JAMA (2007) 297:267–77. 10.1001/jama.297.3.267 17227978

[17] Neoptolemos JP Stocken DD Bassi C Ghaneh P Cunningam D Goldstein D. Adjuvant Chemotherapy With Fluorouracil Plus Folinic Acid *vs* Gemcitabine Following Pancreatic Cancer Resection: A Randomized Controlled Trial. JAMA (2010) 304:1073–81. 10.1001/jama.2010.1275 20823433

[18] Valle JW Palmer D Jackson R Cox T Neoptolemos JP Ghaneh P. Optimal Duration and Timing of Adjuvant Chemotherapy After Definitive Surgery for Ductal Adenocarcinoma of the Pancreas: Ongoing Lessons From the ESPAC-3 Study. J Clin Oncol (2014) 32:504–12. 10.1200/JCO.2013.50.7657 24419109

[19] Neoptolemos JP Palmer DH Ghaneh P Psarelli EE Valle JW Halloran CM. Comparison of Adjuvant Gemcitabine and Capecitabine With Gemcitabinemonotherapy in Patients With Resected Pancreatic Cancer (ESPAC-4): A Multicentre, Open-Label, Randomised, Phase 3 Trial. Lancet (2017) 389:1011–24. 10.1016/S0140-6736(16)32409-6 28129987

[20] Conroy T Hammel P Hebbar M Abdelghani MB Wei AC Raoul J. FOLFIRINOX or Gemcitabine as Adjuvant Therapy for Pancreatic Cancer. N Engl J Med (2018) 379:2395–406. 10.1056/NEJMoa1809775 30575490

[21] Vasile E Vivaldi C Bianco R Lonardi S Di Donato S Brugnatelli S. Randomized Phase 3 Study of Adjuvant Chemotherapy With Folfoxiri Compared to Gemcitabine in Resected Pancreatic Cancer: The “Gruppo Italiano PANCREAS” GIP-2 Study. Abstract book 21th Congress Ital Assoc Med Oncol (2019) 105:1–216. 10.1177/0300891619872589

[22] Tempero MA Reni M Riess H Pelzer U O’Reilly EM Winter JM. APACT: Phase III, Multicenter, International, Open-Label, Randomized Trial of Adjuvantnab-Paclitaxel Plus Gemcitabine (Nab-P/G) *vs* Gemcitabine (G) for Surgically Resected Pancreatic Adenocarcinoma. J Clin Oncol (2019) 37:4000. 10.1093/annonc/mdz247.010

[23] Sinn M Bahra M Liersch T Gellert K Messmann H Bechstein W. CONKO-005: Adjuvant Chemotherapy With Gemcitabine Plus Erlotinib Versus Gemcitabine Alone in Patients After R0 Resection of Pancreatic Cancer: A Multicenter Randomized Phase III Trial. J Clin Oncol (2017) 35:3330–7. 10.1200/JCO.2017.72.6463 28817370

[24] Klinkenbijl JH Jeekel J Sahmoud T van Pel R Couvreur ML Veenhof CH. Adjuvant Radiotherapy and 5-Fluorouracil After Curativeresection of Cancer of the Pancreas and Periampullary Region: Phase III Trial of the EORTC Gastrointestinaltract Cancer Cooperative Group. Ann Surg (1999) 230:776–82. 10.1097/00000658-199912000-00006 PMC1420941 10615932

[25] Rutter CE Park HS Corso CD Lester-Coll NH Mancini BR Yeboa DN. Addition of Radiotherapy to Adjuvant Chemotherapy is Associated With Improved Overall Survival in Resected Pancreatic Adenocarcinoma: An Analysis of the National Cancer Data Base. Cancer (2015) 121:4141–9. 10.1002/cncr.29652 26280559

[26] Hsieh MC Chang WW Yu HH Lu CY Chang CL Chow JM. Adjuvant Radiotherapy and Chemotherapy Improve Survival in Patients With Pancreatic Adenocarcinomareceiving Surgery: Adjuvant Chemotherapy Alone is Insufficient in the Era of Intensity Modulation Radiation Therapy. Cancer Med (2018) 7:2328–233. 10.1002/cam4.1479 PMC6010773 29665327

[27] Hruban RH Goggins M Parsons J Kern SE. Progression Model for Pancreatic Cancer. Clin Cancer Res (2000) 6:2969–72. 10955772

[28] Vogelstein B Kinzler KW. The Multistep Nature of Cancer. Trends Genet (1993) 9:138–41. 10.1016/0168-9525(93)90209-Z 8516849

[29] Makohon-Moore A Iacobuzio-Donahue CA. Pancreatic Cancer Biology and Genetics From an Evolutionary Perspective. Nat Rev Cancer (2016) 16:553–65. 10.1038/nrc.2016.66 PMC5739515 27444064

[30] Waters AM Der CJ. KRAS: The Critical Driver and Therapeutic Target for Pancreatic Cancer. Cold Spring Harb Perspect Med (2018) 8:a031435. 10.1101/cshperspect.a031435 29229669 PMC5995645

[31] Lee KE Bar-Sagi D. Oncogenic KRas Suppresses Inflammation-Associated Senescence of Pancreatic Ductal Cells. Cancer Cell (2010) 18:448–58. 10.1016/j.ccr.2010.10.020 PMC3397918 21075310

[32] Kim JY Hong SM. Precursor Lesions of Pancreatic Cancer. Oncol Res Treat (2018) 41:603–10. 10.1159/000493554 30269131

[33] Tsuda M Fukuda A Takaori K Seno H. Genetics and Biology of Pancreatic Cancer and its Precursor Lesions: Lessons Learned From Human Pathology and Mouse Models. Ann Pancreatic Cancer (2019) 2. 10.21037/apc.2019.07.02

[34] Carrera S Sancho A Azkona E Azkuna J Lopez-Vivanco G. Hereditary Pancreatic Cancer: Related Syndromes and Clinical Perspective. Hered Cancer Clin Pract (2017) 15:9. 10.1186/s13053-017-0069-6 28670351 PMC5490219

[35] Grant RC Selander I Connor AA Selvarajah S Borgida A Briollais L. Prevalence of Germline Mutations in Cancer Predisposition Genes in Patients With Pancreatic Cancer. Gastroenterology (2015) 148:556–64. 10.1053/j.gastro.2014.11.042 PMC4339623 25479140

[36] Wong W Raufi AG Safyan RA Bates SE Manji GA. BRCA Mutations in Pancreas Cancer: Spectrum, Current Management, Challenges and Future Prospects. Cancer Manag Res (2020) 12:2731–42. 10.2147/CMAR.S211151 PMC7185320 32368150

[37] Hu ZI Shia J Stadler ZK Varghese AM Capanu M Salo-Mullen E. Evaluating Mismatch Repair Deficiency in Pancreatic Adenocarcinoma: Challenges and Recommendations. Clin Cancer Res (2018) 24:1326–36. 10.1158/1078-0432.CCR-17-3099 PMC5856632 29367431

[38] Murphy SJ Hart SN Halling GC Johnson SH Smadbeck JB Drucker T. Integrated Genomic Analysis of Pancreatic Ductal Adenocarcinomas Reveals Genomic Rearrangement Events as Significant Drivers of Disease. Cancer Res (2016) 76:749–61. 10.1158/0008-5472.CAN-15-2198 PMC4926317 26676757

[39] Hingorani SR Wang L Multani AS Combs C Deramaudt TB Hruban RH. Trp53R127H and KrasG12D Cooperate to Promote Chromosomal Instability and Widely Metastatic Pancreatic Ductal Adenocarcinoma in Mice. Cancer Cell (2005) 7:469–83. 10.1016/j.ccr.2005.04.023 15894267

[40] Krebs AM Mitschke J Losada ML Schmalhofer O Boerries M Busch H. The EMTactivator Zeb1 is a Key Factor for Cell Plasticity and Promotes Metastasis in Pancreatic Cancer. Nat Cell Biol (2017) 19:518–42. 10.1038/ncb3513 28414315

[41] Rodriguez-Aznar E Wiesmüller L Sainz B Jr Hermann PC. EMT and Stemness-Key Players in Pancreatic Cancer Stem Cells. Cancers (Basel) (2019) 11:1136. 10.3390/cancers11081136 PMC6721598 31398893

[42] Gaianigo N Melisi D Carbone C. EMT and Treatment Resistance in Pancreatic Cancer. Cancers (Basel) (2017) 9:122. 10.3390/cancers9090122 PMC5615337 28895920

[43] Baghban R Roshangar L Jahanban-Esfahlan R Seidi K Ebrahimi-Kalan A Jaymand M. Tumor Microenvironment Complexity and Therapeutic Implications at a Glance. Cell Commun Signal (2020) 1 8:59. 10.1186/s12964-020-0530-4 PMC7140346 32264958

[44] Kikuta K Masamune A Watanabe T Ariga H Itoh H Hamada S. Pancreatic Stellate Cells Promote Epithelial-Mesenchymal Transition in Pancreatic Cancer Cells. Biochem Biophys Res Commun (2010) 403:380–84. 10.1016/j.bbrc.2010.11.040 21081113

[45] Rucki AA Zheng L. Pancreatic Cancer Stroma: Understanding Biology Leads to New Therapeutic Strategies. World J Gastroenterol (2014) 20:2237–46. 10.3748/wjg.v20.i9.2237 PMC3942829 24605023

[46] Palumbo A Jr Da Costa Nde O Bonamino MH Pinto LF Nasciutti LE. Genetic Instability in the Tumor Microenvironment: A New Look at an Old Neighbor. Mol Cancer (2015) 14:145. 10.1186/s12943-015-0409-y 26227631 PMC4521350

[47] Pure E Lo A. Can Targeting Stroma Pave the Way to Enhanced Antitumor Immunity and Immunotherapy of Solid Tumors? Cancer Immunol Res (2016) 4:269–78. 10.1158/2326-6066.CIR-16-0011 PMC5452418 27036971

[48] Steele NG Biffi G Kemp SB Zhang Y Drouillard D Syu L. Inhibition of Hedgehog Signaling Alters Fibroblast Composition in Pancreatic Cancer. Clin Cancer Res (2021) 27(7);2023–37. 10.1158/1078-0432.CCR-20-3715 PMC8026631 33495315

[49] Hwang RF Moore T Arumugam T Ramachandran V Amos KD Rivera A. Cancer-Associated Stromal Fibroblasts Promot Pancreatic Tumor Progression. Cancer Res (2008) 68:918–26. 10.1158/0008-5472.CAN-07-5714 PMC2519173 18245495

[50] Gao Z Wang X Wu K Zhao Y Hu G. Pancreatic Stellate Cells Increase the Invasion of Human Pancreatic Cancer Cells Through the Stromal Cell-Derived Factor-1/CXCR4 Axis. Pancreatology (2010) 10:186–93. 10.1159/000236012 20484957

[51] Clark CE Hingorani SR Mick R Combs C Tuveson DA Vonderheide RH. Dynamics of the Immune Reaction to Pancreatic Cancer From Inception to Invasion. Cancer Res (2007) 67:9518–27. 10.1158/0008-5472.CAN-07-0175 17909062

[52] Javadrashid D Baghbanzadeh A Hemmat N Hajiasgharzadeh K Nourbakhsh NS Lotfi Z. Envisioning the Immune System to Determine its Role in Pancreatic Ductal Adenocarcinoma: Culprit or Victim? Immunol Lett (2021) 232:48–59. 10.1016/j.imlet.2021.02.009 33647329

[53] Leinwand J Miller G. Regulation and Modulation of Antitumor Immunity in Pancreatic Cancer. Nat Immunol (2020) 21:1152–9. 10.1038/s41590-020-0761-y 32807942

[54] Connor AA Denroche RE Jang GH Timms L Kalimuthu SN Selander I. Association of Distinct Mutational Signatures With Correlates of Increased Immune Activity in Pancreatic Ductal Adenocarcinoma. JAMA Oncol (2017) 3:774–83. 10.1001/jamaoncol.2016.3916 PMC5824324 27768182

[55] Yachida S Jones S Bozic I Antal T Leary R Fu B. Distant Metastasis Occurs Late During the Genetic Evolution of Pancreatic Cancer. Nature (2010) 467:1114–7. 10.1038/nature09515 PMC3148940 20981102

[56] Ansari D Bauden M Bergstrom S Rylance R Marko-Varga G Andersson R. Relationship Between Tumour Size and Outcome in Pancreatic Ductal Adenocarcinoma. Br J Surg (2017) 104:600–7. 10.18632/oncotarget.24019 28177521

[57] Kulemann B Rosch S Seifert S Timme S Bronsert P Seifert G. Pancreatic Cancer: Circulating Tumor Cells and Primary Tumors Show Heterogeneous KRAS Mutations. Sci Rep (2017) 7:4510. 10.1038/s41598-017-14870-3 28674438 PMC5495768

[58] Martini V Timme-Bronsert S Fichtner-Feigl S Hoeppner J Kulemann B. Circulating Tumor Cells in Pancreatic Cancer: Current Perspectives. Cancers Basel (2019) 11:1659. 10.3390/cancers11111659 PMC6895979 31717773

[59] Pantel K Speicher MR. The Biology of Circulating Tumor Cells. Oncogene (2016) 35:1216–24. 10.1038/onc.2015.192 26050619

[60] Hasanain A Blanco BA Yu J Wolfgang CL. The Importance of Circulating and Disseminated Tumor Cells in Pancreatic Cancer. Surg Open Sci (2019) 1(2):49–55. 10.1016/j.sopen.2019.08.002 32754693 PMC7391911

[61] Ayres Pereira M Chio IIC. Metastasis in Pancreatic Ductal Adenocarcinoma: Current Standing and Methodologies. Genes Basel (2019) 11:6. 10.3390/genes11010006 PMC7016631 31861620

[62] Grünwald B Harant V Schaten S Frühschütz M Spallek R Höchst B. Pancreatic Premalignant Lesions Secrete Tissue Inhibitor of Metalloproteinases-1, Which Activates Hepatic Stellate Cells *via* CD63 Signaling to Create a Premetastatic Niche in the Liver. Gastroenterology (2016) 151:1011–24. 10.1053/j.gastro.2016.07.043 27506299

[63] Costa-Silva B Aiello NM Ocean AJ Singh S Zhang H Thakur BK. Pancreatic Cancer Exosomes Initiate Pre-Metastatic Niche Formation in the Liver. Nat Cell Biol (2015) 17:816–26. 10.1038/ncb3169 PMC5769922 25985394

[64] Reichert M Bakir B Moreira L Pitarresi JR Feldmann K Simon L. Regulation of Epithelial Plasticity Determines Metastatic Organotropism in Pancreatic Cancer. Dev Cell (2018) 45:696–711.e8. 10.1016/j.devcel.2018.05.025 29920275 PMC6011231

[65] Whatcott CJ Diep CH Jiang P Watanabe A LoBello J Sima C. Desmoplasia in Primary Tumors and Metastatic Lesions of Pancreatic Cancer. Clin Cancer Res (2015) 21:3561–8. 10.1158/1078-0432.CCR-141051 PMC4526394 25695692

[66] Chaika NV Yu F Purohit V Mehla K Lazenby AJ DiMaio D. Differential Expression of Metabolic Genes in Tumor and Stromal Components of Primary and Metastatic Loci in Pancreatic Adenocarcinoma. PloS One (2012) 7:1–10. 10.1371/journal.pone.0032996 PMC3296773 22412968

[67] Aiello NM Bajor DL Norgard RJ Sahmoud A Bhagwat N Minh NP. Metastatic Progression is Associated With Dynamic Changes in the Local Microenvironment. Nat Commun (2016) 7:12819. 10.1038/ncomms12819 27628423 PMC5027614

[68] Connor AA Denroche RE Jang GH Lemire M Zhang A Chan-Seng-Yue M. Integration of Genomic and Transcriptional Features in Pancreatic Cancer Reveals Increased Cell Cycle Progression in Metastases. Cancer Cell (2019) 3 5:267–282.e7. 10.1016/j.ccell.2018.12.010 PMC6398439 30686769

[69] Hasan S Jacob R Manne U Paluri R. Advances in Pancreatic Cancer Biomarkers. Oncol Rev (2019) 13:410. 10.4081/oncol.2019.410 31044028 PMC6478006

[70] Bergquist JR Puig CA Shubert CR Groeschl RT Habermann EB Kendrick ML. Carbohydrate Antigen 19-9 Elevation in Anatomically Resectable, Early Stage Pancreatic Cancer Is Independently Associated With Decreased Overall Survival and an Indication for Neoadjuvant Therapy: A National Cancer Database Study. J Am Coll Surg (2016) 223:52–65. 10.1016/j.jamcollsurg.2016.02.009 27049786

[71] Zhang Y Yang J Li H Wu Y Zhang H Chen W. Tumor Markers CA19-9, CA242 and CEA in the Diagnosis of Pancreatic Cancer: A Meta-Analysis. Int J Clin Exp Med (2015) 8:11683–91. PMC4565388 26380005

[72] Gold DV Gaedcke J Ghadimi BM Goggins M Hruban RH Liu M. PAM4 Immunoassay Alone and in Combination With CA19-9 for the Detection of Pancreatic Adenocarcinoma. Cancer (2013) 119:522–8. 10.1002/cncr.27762 PMC3502643 22898932

[73] Song J Sokoll LJ Pasay JJ Rubin AL Li H Bach DM. Identification of Serum Biomarker Panels for the Early Detection of Pancreatic Cancer. Cancer Epidemiol Biomarkers Prev (2019) 28:174–82. 10.1158/1055-9965.EPI-18-0483 PMC6324978 30333219

[74] Rho SY Lee SG Park M Lee J Lee SH Hwang HK. Developing a Preoperative Serum Metabolome-Based Recurrence-Predicting Nomogram for Patients With Resected Pancreatic Ductal Adenocarcinoma. Sci Rep (2019) 9:18634. 10.1038/s41598-019-55016-x 31819109 PMC6901525

[75] Ankeny JS Court CM Hou S Li Q Song M Wu D. Circulating Tumour Cells as a Biomarker for Diagnosis and Staging in Pancreatic Cancer. Br J Cancer (2016) 114:1367–75. 10.1038/bjc.2016.121 PMC4984454 27300108

[76] Effenberger KE Schroeder C Hanssen A Wolter S Eulenburg C Tachezy M. Improved Risk Stratification by Circulating Tumor Cell Counts in Pancreatic Cancer. Clin Cancer Res (2018) 24:2844–50. 10.1158/1078-0432.CCR-18-0120 29559560

[77] Court CM Ankeny JS Sho S Winograd P Hou S Song M. Circulating Tumor Cells Predict Occult Metastatic Disease and Prognosis in Pancreatic Cancer. Ann Surg Oncol (2018) 25:1000–8. 10.1245/s10434-017-6290-8 PMC5896564 29442211

[78] Poruk KE Blackford AL Weiss MJ Cameron JL He J Goggins M. Circulating Tumor Cells Expressing Markers of Tumor-Initiating Cells Predict Poor Survival and Cancer Recurrence in Patients With Pancreatic Ductal Adenocarcinoma. Clin Cancer Res (2017) 23:2681–90. 10.1158/1078-0432.CCR-16-1467 PMC5407944 27789528

[79] Jaworski JJ Morgan RD Sivakumar S. Circulating Cell-Free Tumour DNA for Early Detection of Pancreatic Cancer. Cancers Basel (2020) 12:3704. 10.3390/cancers12123704 PMC7763954 33317202

[80] Lee B Lipton L Cohen J Tie J Javed AA Li L. Circulating Tumor DNA as a Potential Marker of Adjuvant Chemotherapy Benefit Following Surgery for Localized Pancreatic Cancer. Ann Oncol (2019) 30:1472–8. 10.1093/annonc/mdz200 PMC6771221 31250894

[81] Hussung S Akhoundova D Hipp J Follo M Klar RFU Philipp U. Longitudinal Analysis of Cell-Free Mutated KRAS and CA 19-9 Predicts Survival Following Curative Resection of Pancreatic Cancer. BMC Cancer (2021) 21:49. 10.1186/s12885-020-07736-x 33430810 PMC7802224

[82] Yee NS Zhang S He HZ Zheng SY. Extracellular Vesicles as Potential Biomarkers for Early Detection and Diagnosis of Pancreatic Cancer. Biomedicines (2020) 8:581. 10.3390/biomedicines8120581 PMC7762339 33297544

[83] Ventriglia J Petrillo A Huerta Alváro M Laterza MM Savastano B Gambardella V. Neutrophil to Lymphocyte Ratio as a Predictor of Poor Prognosis in Metastatic Pancreatic Cancer Patients Treated With Nab-Paclitaxel Plus Gemcitabine: A Propensity Score Analysis. Gastroenterol Res Pract (2018) 2018:2373868. 10.1155/2018/2373868 29983708 PMC6015665

[84] Stotz M Gerger A Eisner F Szkandera J Loibner H Ress AL. Increased Neutrophil-Lymphocyte Ratio is a Poor Prognostic Factor in Patients With Primary Operable and Inoperable Pancreatic Cancer. Br J Cancer (2013) 109:416–21. 10.1038/bjc.2013.332 PMC3721392 23799847

[85] Smith RA Bosonnet L Raraty M Sutton R Neoptolemos JP Campbell F. Preoperative Platelet-Lymphocyte Ratio is an Independent Significant Prognostic Marker in Resected Pancreatic Ductal Adenocarcinoma. Am J Surg (2009) 197:466–72. 10.1016/j.amjsurg.2007.12.057 18639229

[86] Jomrich G Gruber ES Winkler D Hollenstein M Gnant M Sahora K. Systemic Immune-Inflammation Index (SII) Predicts Poor Survival in Pancreatic Cancer Patients Undergoing Resection. J Gastrointest Surg (2020) 24:610–8. 10.1007/s11605-019-04187-z PMC7064450 30923999

[87] Kim N Han IW Ryu Y Hwang DW Heo JS Choi DW. Predictive Nomogram for Early Recurrence After Pancreatectomy in Resectable Pancreatic Cancer: Risk Classification Using Preoperative Clinicopathologic Factors. Cancers Basel (2020) 12:137. 10.3390/cancers12010137 PMC7016958 31935830

[88] Martinelli P Carrillo-de-Santa Pau E Cox T Sainz BJ Dusetti N Greenhalf W. GATA6 Regulates EMT and Tumour Dissemination and is a Marker of Response to Adjuvant Chemotherapy in Pancreatic Cancer. Gut (2017) 66:1665–76. 10.1136/gutjnl-2015-311256 PMC5070637 27325420

[89] Collisson EA Sadanandam A Olson P Gibb WJ Truitt M Gu S. Subtypes of Pancreatic Ductal Adenocarcinoma and Their Differing Responses to Therapy. Nat Med (2011) 17:500–3. 10.1038/nm.2344 PMC3755490 21460848

[90] Moffitt RA Marayati R Flate EL Volmar KE Herrera Loeza SG Hoadley KA. Virtual Microdissection Identifies Distinct Tumor- and Stroma-Specific Subtypes of Pancreatic Ductal Adenocarcinoma. Nat Genet (2015) 47:1168–78. 10.1038/ng.3398 PMC4912058 26343385

[91] O’Kane GM Grunwald B Jang G Masoomian M Picardo S Grant RC. GATA6 Expression Distinguishes Classical and Basal-Like Subtypes in Advanced Pancreatic Cancer. Clin Cancer Res (2020) 6:4901–10. 10.1158/1078-0432.CCR-19-3724 32156747

[92] Greenhalf W Ghane P Neoptolemos JP Palmer DH Cox TF Lamb RF. Pancreatic Cancer Hent1 Expression and Survival From Gemcitabine in Patients From the ESPAC-3 Trial. JNCI (2013) 106:djt347. 10.1093/jnci/djt347 24301456

[93] Shin DW Kim MJ Yang SY Lee J Hwang J. Adjuvant Gemcitabine Versus 5-Fluorouracil/Folinic Acid Based on Hent1 Immunostaining in Curative Resected Pancreatic Adenocarcinoma: A Biomaker Stratified Trial. JCO (2019) 37:308–8. 10.1200/JCO.2019.37.4_suppl.308

[94] Randazzo O Papini F Mantini G Gregori A Parrino B Liu DSK. “Open Sesame?”: Biomarker Status of the Human Equilibrative Nucleoside Transporter-1 and Molecular Mechanisms Influencing its Expression and Activity in the Uptake and Cytotoxicity of Gemcitabine in Pancreatic Cancer. Cancers (Basel) (2020) 12(11):3206. 10.3390/cancers12113206 PMC7692081 33142664

[95] Spratlin JL Mackey JR. Human Equilibrative Nucleoside Transporter 1 (Hent1) in Pancreatic Adenocarcinoma: Towards Individualized Treatment Decisions. Cancers (Basel) (2010) 2(4):2044–54. 10.3390/cancers2042044 PMC3840445 24281217

[96] Nicolle R Gayet O Duconseil P Vanbrugghe C Roques J Bigonnet M. A Transcriptomic Signature to Predict Adjuvant Gemcitabine Sensitivity in Pancreatic Adenocarcinoma. Ann Onc (2021) 32:250–60. 10.1016/j.annonc.2020.10.601 33188873

[97] Chan-Seng-Yue M Jaeseung CK Wilson GW Karen NG Figueroa EF O’Kane GM. Transcription Phenotypes of Pancreatic Cancer are Driven by Genomic Events During Tumor Evolution. Nat Genet (2020) 52:231–40. 10.1038/s41588-019-0566-9 31932696

[98] Moore MJ Golstein D Hamm J Figer A Hecht JR Gallinger S. Erlotinib Plus Gemcitabine Compared With Gemcitabine Alone in Patients With Advanced Pancreatic Cancer: A Phase III Trial of the National Cancer Institute of Canada Clinical Trials Group. JCO (2007) 25:1960–6. 10.1200/JCO.2006.07.9525 17452677

[99] Oliveira-Cunha M Newman WJ Siriwardena AK. Epidermal Growth Factor Receptor in Pancreatic Cancer. Cancers Basel (2011) 3:1513–26. 10.3390/cancers3021513 PMC3757375 24212772

[100] Ross A Winter KA Safran H Goodman KA Regine WF Berger AC. Results of the NRG Oncology/RTOG 0848 Adjuvant Chemotherapy Question—Erlotinib+Gemcitabine for Resected Cancer of the Pancreatic Head A Phase II Randomized Clinical Trial. Am J Clin Oncol (2020) 43:173–9. 10.1097/COC.0000000000000633 PMC7280743 31985516

[101] Hoyer K Hablesreiter R Inoue Y Yoshida K Briest F Christen F. A Genetically Defined Signature of Responsiveness to Erlotinib in Early-Stage Pancreatic Cancer Patients: Results From the CONKO-005 Trial. EBioMedicine (2021) 66:103327. 10.1016/j.ebiom.2021.103327 33862582 PMC8054140

[102] Waddell N Pajic M Patch AM Chang DK Kassahn KS Bailey P. Whole Genomes Redefine the Molecular Landscape of Pancreatic Cancer. Nature (2015) 518:495–501. 10.1038/nature14169 25719666 PMC4523082

[103] Golan T Hammel P Reni M Van Cutsem E Maraculla T Hall MJ. Maintenance Olaparib for Germline BRCA-Mutated Metastatic Pancreatic Cancer. N Engl J Med (2019) 381:317–27. 10.1056/NEJMoa1903387 PMC6810605 31157963

[104] Sohal D McDonough S Ahmad SA Gandhi N Beg M Wang-Gillam A. SWOG S1505: Initial Findings on Eligibility and Neoadjuvant Chemotherapy Experience With Mfolfirinox Versus Gemcitabine/Nab-Paclitaxel for Resectable Pancreatic Adenocarcinoma. JCO (2019) 37:414. 10.1200/JCO.2019.37.4_suppl.414

[105] Skau Rasmussen L Vittrup B Ladekarl M Pfeiffer P Karen Yilmaz M Østergaard Poulsen L. The Effect of Postoperative Gemcitabine on Overall Survival in Patients With Resected Pancreatic Cancer: A Nationwide Population-Based Danish Register Study. Acta Oncol (2019) 58:864–71. 10.1080/0284186X.2019.1581374 30905248

[106] Reni M Balzano G Zanon S Zerbi A Rimassa L Castoldi R. Safety and Efficacy of Preoperative or Postoperative Chemotherapy for Resectable Pancreatic Adenocarcinoma (PACT-15): A Randomized, Open-Label, Phase 2-3 Trial. Lancet Gastroenterol Hepatol (2018) 3(6):30094–3. 10.1016/S2468-1253(18)30081-5 29625841

[107] Roland CL Katz MHG Tzeng C-WD Lin H Varadhachary GR Shroff R. The Addition of Postoperative Chemotherapy is Associated With Improved Survival in Patients With Pancreatic Cancer Treated With Preoperative Therapy. Ann Surg Oncol (2015) 22:1221–8. 10.1245/s10434-015-4854-z PMC5192562 26350371

[108] Van Roessel S van Veldhuisen E Klompmaker S Janssen QP Abu Hilal M Alseidi A. Evaluation of Adjuvant Chemotherapy in Patients With Resected Pancreatic Cancer After Neoadjuvant FOLFIRINOX Treatment. JAMA Oncol (2020) 6:1–8. 10.1001/jamaoncol.2020.3537 PMC7489392 32910170

[109] A Phase 0, Pre-Operative, Window-Of-Opportunity Study to Assess Gene Expression in Patients With Resectable, Locally Advanced, or Metastatic Pancreatic Cancer (NEOPANC-01). Available at: https://pancreaticcancercanada.ca/press-release-neopancone-clinical-trial-launch/.

[110] Luchini C Brosens L Wood D Chatterjee D Shin J Sciammarella C. Comprehensive Characterisation of Pancreatic Ductal Adenocarcinoma With Microsatellite Instability: Histology, Molecular Pathology and Clinical Implications. Gut (2021) 70:148–56. 10.1136/gutjnl-2020-320726 PMC7211065 32350089

[111] Le DT Uram JM Wang H Bartlett BR Kerberling H Eyring AD. PD-1 Blockade in Tumors With Mismatch-Repair Deficiency. N Engl J Med (2015) 372:2509–20. 10.1056/NEJMoa1500596 PMC4481136 26028255

[112] Marabelle A Le DT Ascierto PA Di Giacomo AM De Jesus-Acosta A Delord JP. Efficacy of Pembrolizumab in Patients With Noncolorectal High Microsatellite Instability/Mismatch Repair-Deficient Cancer: Results From the Phase II KEYNOTE-158 Study. JCO (2020) 38:1–10. 10.1200/JCO.19.02105 PMC8184060 31682550

[113] Pompella L Tirino G Pappalardo A Caterino M Ventriglia A Nacca V. Pancreatic Cancer Molecular Classifications: From Bulk Genomics to Single Cell Analysis. Int J Mol Sci (2020) 21:2814. 10.3390/ijms21082814 PMC7215357 32316602

[114] Pancreatic Tumor Cell Vaccine (GVAX), Low Dose Cyclophosphamide, Fractionated Stereotactic Body Radiation Therapy (SBRT), and FOLFIRINOX Chemotherapy in Patients With Resected Adenocarcinoma of the Pancreas. Available at: https://www.clinicaltrials.gov/ct2/show/NCT01595321.

[115] Samaras p Tusup M Nguyen-Kim TDL Seifurt B Bachmann H von Moos R. Phase I Study of a Chloroquine-Gemcitabine Combination in Patients With Metastatic or Unresectable Pancreatic Cancer. Cancer Chemother Pharmacol (2017) 80:1005–12. 10.1007/s00280-017-3446-y 28980060

[116] Karasic TB O’hara M Loaiza-Bonilla A Reiss KA Teitelbaum UR Borazanci E. Effect of Gemcitabine and Nab-Paclitaxel With or Without Hydroxychloroquine on Patients With Advanced Pancreatic Cancer A Phase 2 Randomized Clinical Trial. JAMA Oncol (2019) 5:993–8. 10.1001/jamaoncol.2019.0684 PMC6547080 31120501

[117] Janssen QP Buettner S Suker M Beumer BR Addeo P Bachellier P. Neoadjuvant FOLFIRINOX in Patients With Borderline Resectable Pancreatic Cancer: A Systematic Review and Patient-Level Meta-Analysis. J Natl Cancer Inst (2019) 111:782–94. 10.1093/jnci/djz073 PMC6695305 31086963

[118] Suker M Beumer BR Sadot E Marthey L Faris JE Mellon EA. FOLFIRINOX for Locally Advanced Pancreatic Cancer: A Systematic Review and Patient-Level Meta-Analysis. Lancet Oncol (2016) 1 7:801–10. 10.1016/S1470-2045(16)00172-8 PMC5527756 27160474

[119] Cascinu S Berardi R Bianco R Bilancia D Zaniboni A Ferrari D. Nab-Paclitaxel (Nab) Plus Gemcitabine (G) Is More Effective Than G Alone in Locally Advanced, Unresectable Pancreatic Cancer (LAUPC): The GAP Trial, a GISCAD Phase II Comparative Randomized Trial. Ann Oncol (2019) 30:253–v254. 10.1093/annonc/mdz247.001

[120] Philip PA Lacy J Portales F Sobrero A Pazo-Cid R Manzano Mozo JL. Nab-Paclitaxel Plus Gemcitabine in Patients With Locally Advanced Pancreatic Cancer (LAPACT): A Multicentre, Open-Label Phase 2 Study. Lancet Gastroenterol Hepatol (2020) 5:285–94. 10.1016/S2468-1253(19)30327-9 31953079

[121] Mukherjee S Hurt CN Bridgewater J Falk S Cummins S Wasan H. Gemcitabine-Based or Capecitabine-Based Chemoradiotherapy for Locally Advanced Pancreatic Cancer (SCALOP): A Multicentre, Randomised, Phase 2 Trial. Lancet Oncol (2013) 14:317–26. 10.1016/S1470-2045(13)70021-4 PMC3620899 23474363

[122] Hammel P Huguet F van Laethem JL Goldstein D Glimelius B Artru P. Effect of Chemoradiotherapy *vs* Chemotherapy on Survival in Patients With Locally Advanced Pancreatic Cancer Controlled After 4 Months of Gemcitabine With or Without Erlotinib: The LAP07 Randomized Clinical Trial. JAMA (2016) 315:1844–53. 10.1001/jama.2016.4324 27139057

[123] Williet N Petrillo A Roth G Ghidini M Petrova M Forestier J. Gemcitabine/Nab-Paclitaxel Versus FOLFIRINOX in Locally Advanced Pancreatic Cancer: A European Multicenter Study. Cancers (2021) 13:2797. 10.3390/cancers13112797 34199796 PMC8200096

[124] Versteijne E Suker M Groothuis K Akkermans-Vogelaar JM Besselink MG Bonsing BA. Preoperative Chemoradiotherapy Versus Immediate Surgery for Resectable and Borderline Resectable Pancreatic Cancer: Results of the Dutch Randomized Phase III PREOPANC Trial. JCO (2020) 38:1763–73. 10.1200/JCO.19.02274 PMC8265386 32105518

[125] Lee JS Rhee TM Pietrasz D Bachet JB Laurent-Puig P Kong SY. Circulating Tumor DNA as a Prognostic Indicator in Resectable Pancreatic Ductal Adenocarcinoma: A Systematic Review and Meta-Analysis. Sci Rep (2019) 18;9(1):16971. 10.1038/s41598-019-53271-6 PMC6861312 31740696

[126] Hewitt DB Nissen N Hatoum H Musher B Seng J Coveler AL. A Phase III Trial of Chemotherapy With or Without Algenpantucel-L (HyperAcute-Pancreas) Immunotherapy in Subjets With Bordeline Resectable or Locally Advance Unresectable Pancreatic Cancer. Ann Sur (2020). 10.1097/SLA0000000000004669 33630475

[127] Hardacre JM Mulcahy M Small W Talamonti M Obel J Krishnamurthi S. Addition of Algenpantucel-L Immunotherapy to Standard Adjuvant Therapy for Pancreatic Cancer: A Phase 2 Study. J Gastrointest Surg (2013) 17:94–100; discussion p. 100-1. 10.1007/s11605-012-2064-6 23229886

[128] Th-1 Dendritic Cell Immunotherapy Plus Standard Chemotherapy for Pancreatic Adenocarcinoma (DECIST). Available at: https://clinicaltrials.gov/ct2/show/NCT04157127.

[129] Pooled Mutant KRAS-Targeted Long Peptide Vaccine Combined With Nivolumab and Ipilimumab for Patients With Resected MMR-P Colorectal and Pancreatic Cancer . Available at: https://clinicaltrials.gov/ct2/show/NCT0411708707/s11605-012-2064-6.

[130] Prognostic Role of Circulating Tumor DNA in Resectable Pancreatic Cancer. Available at: https://clinicaltrials.gov/ct2/show/NCT04246203.

